# Massachusetts Dental Society

**Published:** 1902-12

**Authors:** 


### MASSACHUSETTS DENTAL SOCIETY.

The thirty-eighth annual meeting of the Massachusetts Dental Society was held at Hotel Brunswick, Boston, Mass., June 4 and 5, 1902. The meeting was called to order by Vice-President Andrew J. Flanagan, of Springfield, Mass.

*Vice-President Flanagan.*—The next business in order on our programme is the annual address of our president. The address of the president may amount to much or little, according to the matter it contains. It has been my pleasure for several years to be a member of both the Northeastern Association and the Massachusetts Dental Society, and it seems to me that one need be only honest and straightforward and faithful in his attendance at these meetings, and he will hear something of vital interest and advantage. This applies to every one of us, and I can say for the president of this society that in him we have had a faithful worker, one who has done what may be termed "scrub" work, and who has been an honor to our Society. I, therefore, esteem it an honor as well as a great pleasure to introduce to you the president, Dr. Frederick S. Faxon, of Brockton, Mass.

PRESIDENT'S ADDRESS.

"Expositions are the time-keepers of progress. They record the world's advancement, they stimulate the energy, enterprise, and intellect of the people, and quicken human genius. They open the mighty storehouses of information to the student.

"Every exposition, great or small, has helped to some onward step. Comparison of ideas is always educational, and as such instructs the brain and hand of man."

You will all probably recognize these as the words with which our late honored President McKinley opened his last speech, and where could they be more appropriately applied than in our own midst?

"Comparison of ideas is always educational, and as such instructs the brain and hand of man."

These last words answer in full, without argument, the question of that member of the dental profession who, when it is suggested he join a dental society, says, "Why should I? What do *I* get out of it?" But, dental brethren, in spite of the fact that this is the attitude of far too many in the profession, it is too narrow for us to dwell upon for a moment. At no time in the history of dentistry has there been more good words said in favor of our association at dental meetings than in the past two years, and this from the true representative men in the profession.

Only as late as April, 1902, in one of the good editorials written on this subject, it is stated "that it cannot be denied that the greatest achievements in dentistry have been usually given to the world by first announcement at a dental meeting." Therefore, it is but just to declare that it is the dental society which is the foundation and corner-stone of dental progress. It is truly said, "The church or nation is what the people in it make it." So with the State dental society, which represents the condition and standing of the profession in its respective State.

The State society is the one to mingle with the rest of the world, to represent the profession of the State in all dental matters in and out of the State, to co-operate with other similar bodies in the general advancement of the profession, *not* for the benefit of the individual dentist particularly (although in no other manner can he get so much individual benefit), but for the benefit of humanity at large, the dentist included. He who is seeking happiness (a condition without limitation) by catering to his own selfish desires will be sadly disappointed by receiving in exchange only temporary pleasure, whose certain limitations too often end in disaster.

The purpose of the State society should be looked upon from a broader and nobler stand-point than that it holds a vaudeville entertainment once a year for the benefit of its members. It is the medium through which the dental progress of the world should come. It should stand for the elevation and advancement of the profession for the benefit of humanity.

There *is* a general desire to elevate and make more of our dental societies, but all are not agreed as to the methods to adopt. There is one vast movement forward in dental education, the progress in compatibility between the examiners and faculties, the unanimous extension of the college course, and one is becoming so dependent on the other as to take the attitude of a great combination, of which the State society is an important factor and cannot be dispensed with. In it should originate the different committees to look after legislation,—inspire good legislation and antagonize bad; something must be done to take the responsibility of prosecution from the general dentist. It should look after the oral hygiene of the State by examination of school-children's teeth. The State society should lead in the protection of the public from such institutions as St. Luke's Hospital. The grand movement going on in the Associated Charities with the aid of our valued and esteemed professional brother Dr. Hopkins, in dental attention to the poorer classes, should have emanated from the State society. No one dentist may take upon himself (for obvious reasons) the enlightenment of the public in regard to the care of the teeth so far as it concerns his profession, but an association of dentists in any community may, and ought to do so. The State society should hold the tiller of State board candidates and keep the standard at the proper elevation. This takes it out of any sect, class, or politics. It should lead in the important matter of interstate reciprocity. The proposed amendment to the Ohio dental law, providing that upon the unanimous vote of the State board, said board may excuse a licentiate from some other State, being the best yet proposed, I would advise this Society to take action on it at this meeting. It should form district societies in places where interest is shown, and local societies are being formed for the self-same purpose.

I will not detain you longer on details, as my purpose is only to give an idea of the attitude and purpose of the State society. With a proper committee as a head and a good substantial fund to bind us together, so that we will not be living from hand to mouth, this work can be carried on to better advantage; there would be more interest, something tangible to work for. Our Society must assume an attitude in which we will take pride.

Mr. Lewis Nixon, a wise and able leader, says, "It is the duty of every young man to take an active part in politics, as much his duty as it is that he work for a living."

The people are the government, the conditions under which we live are entirely of our own making. Why not also apply this same idea to our State dental societies? The condition of dental matters is entirely of our own making, yet this is seemingly realized by only a few. To the extent that we are negligent, thoughtless, and selfish, is dental progress held back from attaining that position of which we, in our thinking moments, are justly proud.

There have been such beautiful words said to you, such splendid suggestions by former presidents, that there seems but little left for me; in fact, nothing can be added. I could only say them over again, depending on the power of repeated suggestion to change these good words into action for the benefit of all concerned. Because something did not explode when they were uttered does not mean they did not have their good effect; they took root somewhere. Ideas have been given us which, if carried out, would have developed us to a degree of perfection hard to realize. And still we are agitating the question of how to increase membership and interest in dental matters and dental progress generally. Now, to bring matters down to a local issue, which is the main object of this address.

Out of two hundred and twenty-seven towns and cities in the State of Massachusetts, only sixty-two are represented in this Society; in twelve cities of over ten thousand population, we cannot claim a member. Lowell, with upward of ninety thousand, sends us no representative, but has formed, in January, 1902, a local society, whose president is a member of the Northeastern District, showing the presence of a spark of interest, which only needed a little fanning. This should be done by the State Society, and I have reason to believe would have been successful in this case in locating a district society. In Somerville it is the same, forming a local society February 10, 1902, for just the purposes State and district societies stand for. We have four members there out of twenty eligible dentists, two of whom are on the executive committee of the local society. Lynn has also formed a local society. Haven't they been waiting for the State Society to act? Occurring at this late date would suggest as much. Now, this work going on right under the nose of the State Society, so to speak, suggests that there is a desire for association among dentists, and that the attitude and purposes of the district society are not generally understood, and a clear and lucid explanation should be sent to every member of our Society. Since dividing the Society into districts in 1895, from a seeming loss of interest, two districts, the North and South Metropolitan, have joined, calling themselves the Metropolitan, absorbing the Northeastern; and the Southeastern District for a time was in an anæmic condition, but, by proper application of medicine, gained in vitality and is all right to-day, with a live membership of thirty-seven in good standing, ten being admitted last year. The Central District with no particular interest in local meetings should be admired for keeping its business in relation to the parent society so well up to the mark, only one member being in arrears for dues, fourteen young practitioners joining this year, full of enthusiasm for the fall campaign, so reported.

It is superfluous to say anything about the Valley District. It is all right, always has been all right, and every one knows it is all right. Eight new members have been elected the past year and one lost by resignation, and the State Society has lost one honorary member in this district. It contains ninety-seven per cent. of the eligible men in the district, and the State Society should be proud of this auxiliary. The Western District is in better shape to-day than ever. There is some trouble in collecting the dues, as the society has been neglected, with the result that several members got in arrears for large amounts, but the new treasurer feels that he can report a clean sheet in a short time. Here we see the value of an interested officer. It would not take one-tenth the interest and time that this laxity causes to properly care for affairs while in good condition.

There has been a renovation in the Western District, leaving twenty-three members in good standing; seven are suspended for arrears in dues, one removal from town, and death has claimed one. What we need is a fan kept in the State Society in the shape of a committee as a head to all details, to see that no flame gets so low as to flicker.

The Metropolitan District from a financial stand-point is our great hold. Its business department stands at the head. It has officers who take pride in attending to their duty, and in detail its affairs are kept up to the mark. It has a membership list of nearly two hundred in good standing, and in comparison with last year's list in the programme shows the addition of thirty new members. So far as its direct bearing on the parent society is concerned, it deserves honorable mention. It holds interesting and well conducted meetings, but as a district society it is a failure in this respect; the district covers too much territory; less than one-third of its members ever attend the meetings, and in all probability the majority do not realize that they belong to anything but the State Society.

The Metropolitan should be subdivided, after due thought as to the best method, aided by experience.

In dividing the State Society in 1895 the correct method was pursued, paying attention to railroad conveniences; but as we see the interest spring up in different cities for the self-same purpose we represent, and where districts were previously, would not the proper attention make them our district societies? There are reasons for believing this.

The importance of good secretary work is shown when you wish to look up any particular proceedings at a particular meeting, change in by-laws, etc. This has been proved in the last year.

The importance of a good treasurer is shown by the lax condition into which the Northeastern, Southeastern, North Metropolitan, and Western Districts were allowed to drop, and the manner in which the Central District and the superfluous part of the Metropolitan Districts are held together and bound to the parent society without any seeming social interest. In some districts the secretary must bear his share of criticism. Now, when I am speaking of inattention to business, laxity of interest by officers, etc., please do not interpret me as taking a pessimistic view of matters and conditions, for nothing is farther from my intention. We were never in better condition, nor has the outlook ever been brighter. It is my intention to deal with fact rather than sentiment. I desire at all times to be suggestive rather than positive or conclusive.

I wish to convey the idea that the progress and success of the society depends mainly on the interest and faithfulness of its officers. There *must* be *three* live men in each district society,—secretary, treasurer, and chairman of the executive committee, and in any district where you cannot get these you can be absolutely certain of a withered limb of the society. An interested officer will, as the year goes by, see many ways by which the details of the society work can be improved; things suggest themselves at times which, if jotted down, can be brought up at the right moment with much benefit. This in detail is what constitutes interest,—a little thought outside of routine work.

These conditions are alluded to principally to show the danger present of going back to former conditions, and where the State Society would have but one district,—the Metropolitan,—or, in fact, be where it was in 1894 before the division. In 1894 the Society numbered about one hundred active members, increasing in 1901 to three hundred, and to-day we have a membership list of three hundred and sixty in good standing.

Do not let us undo the hard and valuable work done in 1895 by the able officers in dividing the Society into districts. *No,* members of the Massachusetts Dental Society, do not let the districts be wiped out. The interest is there more than ever to-day, but they must have a head, and the machinery must be oiled to run smoothly, and there will be no trouble with the existing plan.

No corporation, institution, business, or society can run successfully without proper attention to business details. The department store principle is correct; if the head of a department is not attending to it properly, he is dismissed as a bad link in the chain; the district society is one of the departments of the whole, and a committee should be chosen as a head to the whole business to keep run of affairs. This is what the president is supposed to do, but I do not believe there are exceptions enough among us who would or could give time to attend to this,—certainly not without help. There should be a head to the districts to keep them up to the mark, composed of men who have the interest and welfare of the whole Society at heart. The officers must feel there is some one to be responsible to, some one to criticise if matters are not attended to.

This will be an inspiration for those who hold the positions to fill them faithfully. The committee spoken of, adding the suggestion of starting the fund previously mentioned, would, it seems to me, give a substantial foundation to our organization which would bind us together more firmly.

This committee should see that district officers are doing their duty according to the By-Lays, and guard against any lack of interest that will interfere with State Society affairs, and should be chosen for their ability and interest to attend to these matters; not as severe a task as supposed on first thought, but simply a matter of attending to it. This committee's work is best performed when it has nothing to do, showing a good condition of affairs.

Article III., Section 6, of the State Constitution and By-Laws says that the "District Treasurer shall send a report approved by the Board of Censors of his society of receipts and disbursements during the year, also showing financial condition of said society. In case the District Treasurer shall neglect to make his return as herein provided, he shall be liable to be proceeded against according to law for the whole amount of assessments charged to him on his list."

Was this put in for a purpose, or simply for the sake of saying something? I am willing to give the officers who drew up this section the credit of having the intelligence of knowing what they were doing, and I have a great deal of respect for their ability, and believe it was meant for just what it says. But who is there to proceed against a district treasurer if he does not turn in dues for all members? No one. You will say it is the president's duty. Had this been realized, the lax condition of districts in the past few years would not have occurred, and that is just why we should have this committee with authority. There has been some question as to whether it means that a district treasurer is responsible for just what dues he collects, or for dues of all members collected or not. It is for all members, and it is correct, unreasonable as it seems at first thought.

Article II., Section 2, of the District By-Laws says, "The name of any member who may be in arrears for two years shall, upon vote of the society, be stricken from the roll of membership," and so on.

Now, if the district treasurer attended to his duty according to the By-Laws (and it is a discredit to himself and the society if he accepts the position and does not), there would be no delinquent members for him to be responsible for, only for dues collected. The By-Laws are not only fair, but sensible, as they make a treasurer responsible not only for that money in his possession, but push him to collect or report for action if the society demands it.

The district society is not an independent society, as many have the idea; it is as much a part of the whole as a table leg is a part of the table. It is not obliged to have any dues aside from the State Society. It has a treasurer to collect annual dues of two dollars for the Massachusetts Dental Society; officers called a Board of Censors, to attend to the membership list, investigating the character of applicants, etc.; and a secretary for the usual duties. They must hold a meeting at least twice a year, at which the five officers may constitute a quorum. It is supposed that the districts will hold meetings for social advancement, the reading of essays, clinics, and to increase the interest more generally throughout the State than under the old conditions; this was one of the objects of the division, but not mandatory by constitution, but optional with the members in the district. The fact that they do not do this does not make the society defunct. Therefore, the whole cause of any district society's becoming defunct, as some erroneously say, lies with the district officers, treasurer *principally,* in not collecting dues or reporting delinquents to be acted on; with the executive committee and secretary next; and also the members not understanding the attitude of the district society,—that it is the door through which they must pass to be a member of the Massachusetts Dental Society. Too much dependence has been placed on the district societies being independent societies, with the idea that if they lacked interest in the social side, all must be dropped. The district society is not an independent society, but part of the combination, and a screw loose in one affects the whole. All *officers* should realize this if all members do not. Investigation reveals the fact that what we term delinquents, with exceptions too few to notice, are not in arrears from any intentional neglect on their part, or lack of interest in our State Society work. Their willing and ready response to a proper appeal even to an amount approaching an unnecessary hardship shows that the members of the Massachusetts Dental Society are men of character, and with an interest sufficient to inspire an interested officer and to warrant success in our work as a State society.

Now, if my words savor of pessimism, it is far from my intention. Our State Society is most certainly on the upward trend. Part of the duties of a State society previously mentioned have already been adopted by us, but there is a missing link in the chain, or a weak one which needs strengthening. The correct listing of members in good standing with details this last year is a grand work for the Society, as it places the members where they can be approached when needed for the advancement and carrying on of the Society's work, and the one who carried it through deserves great credit.

The steady progress in society work throughout the country and the increase in membership shows that a better educated class is constantly coming among us. As dentists are developing in education, they are craving association. One-fifth of the profession is engaged in society work; to-day there are over one hundred society organizations and more interest in society work than ever. The country is overflowing with the young practitioner eager for association, whose interest needs but a tickling with the smallest feather to make him one of us. But let us beware that the standard and interest in our association when he does join is not disappointing.

*Vice-President Flanagan.*—The president's address is now before you for discussion.

DISCUSSION.

*Dr. J. T. Paul.*—There is one article which pleased me as much as anything, and that is the report of the return of the district treasurers. I am supposed to know every member in the Society. If the district treasurer does not make a return, I do not see how I am to know that. A recent vote of the Society reads thus: "must keep a correct list of the district society's members, recording each member's indebtedness to the society." I received last fall, ten days before the fall meeting, reports of the treasurer, giving amounts of the individual indebtedness of each member of the district society. You can see from these lists I have here how well that has been carried out. The Metropolitan reports one hundred and ninety-one, but in not one of them have I received the full amount. They make their returns and include in them some of the arrearages. The South District comes in with thirty-seven, but they have made the same return, and several of them are two and three years in arrears. The Central District is behind on thirty-five members. The Valley District has a membership of fifty-five, but has made return for twenty-seven and one-half. The Western District reports a membership of twenty-three, but has made no return whatever. I have been told since I came here that the treasurer gave his check and report to another member, and he came away and forgot it. Now, if I am to have these lists correct, it would be well, when the district treasurers are sending funds to me, to send me a list of members whose yearly dues are paid. This will be of great assistance to me, and without it I can do nothing. The president has read Article III., Section 6, of the Constitution. I would like the treasurers, when they send money, to also send a list of the members for whom the money pays and for what dues. Last year I received from some of the treasurers only a note stating that paid for this year and last year. This year I received some who have paid up to the annual meeting of 1902. It seems to me that this part of the president's address should be brought especially before your attention, and see that the district treasurers look out for themselves, and I will look out for my own part of the work.

*Dr. George A. Maxfield.*—I want to commend the president's address. It shows that he has studied carefully into the condition of affairs and has presented them in an excellent light before us. It also sets a mark for the incoming president to attain to. In the past there has been serious criticism, with some grounds for it, on the addresses of some of the presidents. The greatest thing that troubles me is the lack of business methods throughout the whole State. This association is a chartered organization. We have a constitution and by-laws, and these should be lived up to to the very letter. It is only thus, as we see the needs and requirements, that they can be maintained and brought to a better working basis. The president has recommended that a board of councillors be appointed. I think this an excellent idea. It is but right that the treasurers should make reports, and as the dues from each member are legal debts, they should be collected, but it should be done in a more business-like method than it has been done in the past. I hope these recommendations of the president will be adopted.

*Dr. J. T. Paul.*—I omitted to say, coming back to that article, the treasurer is supposed to make a report in connection with his funds. Now, this year I have received none whatever except from the Metropolitan Society. I know nothing whatever of the condition of any of the districts except the Metropolitan.

*Dr. J. K. Knight.*—I would like to inquire who has the right to terminate membership in the State Society? Whether the State Society or the district society has that right.

*Vice-President Flanagan.*—I would ask the president to answer that question.

*President Faxon.*—I will answer that question for Dr. Knight because I expected it to come up at this meeting. If I understand your question at all, it is in regard to the making and unmaking of members. As I said before, I expected the question to come up here, and so I got advice on it. All active members of this Society are made and unmade in the district societies; the dues are all paid there, and the district society has the whole power to make and unmake the members.

*Dr. J. K. Knight.*—One further question: Can that membership in the State Society be terminated on different grounds, or must the reasons be uniform?

*President Faxon.*—Each district society is privileged to make its own by-laws where they do not conflict with the constitution and by-laws of the State Society. If there is any by-law in regard to membership which conflicts with the by-laws of the State Society, then there is a chance for your argument. If there is *not,* they are all supposed to be the same. If there has been any amendment, anything of that nature, brought before the district society, I know nothing of it.

*Dr. J. K. Knight.*—This is the point I want to bring out. I do not think that any of us want that one district society should set up a limit for the termination of membership which does not exist in the other district societies. The thing should be made uniform. Members coming into the district societies have certain legal rights. I do not believe that one district society has the right to terminate membership for non-payment of dues after three or six months while another district society allows its members two years. That is the point.

*President Faxon.*—I know of nothing which gives one district the privilege over another in regard to the termination of membership. I would rule that all district societies are alike, unless there has been some amendment.

*Dr. J. K. Knight.*—If you will call for the records of the Metropolitan District, you will find that membership is terminated in six months, while the other districts have two years. It seems to me a little unjust. Many of the older members come only to the annual meeting, and if they are to be dropped out in six months' time, many of these older and valuable members will be dropped and you will lose them.

*President Faxon.*—That is an amendment of the district by-laws. How does that interfere with the State Society's by-laws?

*Dr. J. W. Bailey.*—The gentleman refers to the amendment to the constitution of the Metropolitan District which was passed at our annual meeting. At that time a point of order was raised that the vote was illegal, but the chairman ruled that that society, and each society, was a law unto itself, and that there is nothing in the State Society's constitution which says that a district society shall not make its own laws in that respect, and it is absurd, with all respect to my friend here, to give any society the privilege of making members and not give that society the privilege of unmaking memberships. We are all citizens of the United States, not less so, no matter where we live. In regard to losing valuable members, I do not believe we will lose any valuable members by shortening the time. We did drop one valuable member, but he has come back, and I think we have all had a valuable lesson. I contend, Mr. President, that the Metropolitan Society has a legal right to pass any laws, by-laws, or amendments to the by-laws, just so far as they do not conflict with the constitution and by-laws of the State Society. Each society is a law unto itself. I think any lawyer will tell you that is right.

*Vice-President Flanagan.*—Gentlemen, is there any further discussion on this paper? If not, I am going to overstep my bounds a little bit and say that it is really a necessity that some action be taken. I am, therefore, going to ask that some member recommend that a committee be appointed for the consideration of the President's Address. I therefore ask that some member move this.

*Dr. G. A. Maxfield.*—I move that a committee of three be appointed to consider the President's Address and report at the next meeting of the Councillors.

Motion seconded.

*Vice-President Flanagan.*—Gentlemen, you have heard the motion. Is there anything to be said on the question?

*Dr. R. R. Andrews.*—I want to inquire if this means that we shall have a report from that committee during the present session?

*Vice-President Flanagan.*—It will come up under "unfinished business."

Motion put and carried.

*Vice-President Flanagan.*—I will appoint Dr. Maxfield, of the Valley District, Dr. Paul, of the Metropolitan District, and Dr. McLaughlin, of the Western District.

(To be continued.)

